# Abstract of Meteorological Observations for May, 1856, and for the Spring Quarter Ending May 31st, 1856, Made at Philadelphia, Pa.

**Published:** 1856-07

**Authors:** James A. Kirkpatrick


*Abstract of Meteorological Observations for May, 1856, and for the Spring quarter ending May 31st, 1856, made at Philadelphia, Pa. Latitude 39° 57′ 28″ N., Longitude 75° 10′ 40″ W. from Greenwich.*

By Prof. James A. Kirkpatrick.

| 1856. May. | Barometer. Daily Mean | Barometer. Mean Daily Range. | Thermom. Daily Mean | Thermom. Mean Daily Range | Rel. Humid. 2 P.M. | Force of Vapor 2 P.M. | Dew Point 2 P.M. | Rain | Prevailing Winds. | Remarks. |
|---|---|---|---|---|---|---|---|---|---|---|
| | Inches. | Inches. | Deg. | Deg. | Hunds. | Inches. | Deg. | Inches. | Points. | |
| 1 | 30.076 | .113 | 44.0 | 19.5 | 92 | .265 | 41.8 | 1·262 | NE. | *Raining* all day. *Barometer highest* 30.147. |
| 2 | 29.669 | .407 | 53.5 | 10.5 | 75 | .350 | 49.2 | 0.117 | NNE. | Cloudy; *rain* morning and night. |
| 3 | 29.649 | .055 | 48.7 | 7.2 | 82 | .291 | 44.2 | 0.040 | E. | From 11, A.M. to 10, P.M. *drizzling.* |
| ● 4 | 29.745 | .096 | 54.2 | 5.5 | 34 | .176 | 31.3 | | NW. | Clear. |
| 5 | 29.969 | .224 | 53.7 | 0.8 | 40 | .215 | 36.4 | | NW. | Clear. |
| 6 | 30.065 | .134 | 57.7 | 4.0 | 38 | .228 | 37.9 | | (Var.) | Morning clear; afternoon and evening cloudy. |
| 7 | 29.867 | .198 | 49.0 | 8.7 | 82 | .309 | 45.8 | 0.438 | (Var.) | *Rain* all day, *drizzling.* |
| 8 | 29.658 | .209 | 46.7 | 2.3 | 85 | .285 | 43.7 | 0.050 | NE. | Cloudy; *rain drizzling* all day. |
| 9 | 29.645 | .018 | 49.7 | 3.0 | 83 | ·322 | 46.8 | 0.017 | N. | Cloudy; morning, afternoon and night, *drizzling rain.* |
| 10 | 29.630 | .015 | 50.0 | 3.7 | 64 | .268 | 42.2 | | NW. | Cloudy. |
| ☽ 11 | 29.640 | .010 | 66.2 | 16.2 | 32 | .268 | 42.1 | | NW. | Clear. |
| 12 | 29.732 | .092 | 73.3 | 7.2 | 25 | .268 | 42.1 | | (Var.) | Clear. |
| 13 | 30.009 | .278 | 62.2 | 11.8 | 49 | .315 | 46.3 | | NE. | Cloudy. |
| 14 | 30.078 | .080 | 65.5 | 15.0 | 50 | .430 | 54.8 | | S. | Morning and afternoon cloudy; evening clear. |
| 15 | 30.006 | .072 | 72.5 | 7.0 | 42 | .388 | 51.9 | | S. | Morning and afternoon cloudy; evening clear. |
| 16 | 29.888 | .118 | 69.7 | 2.8 | 56 | .483 | 58.0 | 0·040 | S. | Cloudy; 4, P.M. to 10, P.M. *drizzling rain.* |
| 17 | 29.823 | .065 | 73.3 | 3.7 | 35 | .322 | 46.9 | | (Var.) | Cloudy. |
| 18 | 29.725 | .099 | 70.7 | 3.3 | 42 | .408 | 53.4 | | E. | Morning and afternoon cloudy; evening clear. |
| ○ 19 | 29.672 | .053 | 70.7 | 3.3 | 47 | .418 | 54.0 | | SW. | Morning fog; afternoon cloudy; evening clear. |
| 20 | 29.596 | .081 | 73.7 | 3.0 | 30 | .340 | 48.4 | | SW. | Morning fog; afternoon cloudy; evening clear. |
| 21 | 29.794 | .198 | 71.0 | 5.3 | 19 | .149 | 27.4 | | NW. | Clear. Mosquitoes appear. |
| 22 | 29.991 | .196 | 67.3 | 4.3 | 24 | .193 | [illegible] | | NW. | Clear. |
| 23 | 29.936 | .057 | 73.3 | 6.0 | 49 | .435 | 55.1 | | SW. | Clear. |
| 24 | 29.717 | .219 | 78.2 | 4.8 | 50 | .566 | 62.5 | | SW. | Morning and afternoon cloudy; evening clear. *Thermometer highest* 83.5° |
| 25 | 29.690 | .132 | 64.7 | 13.5 | 25 | .176 | 31.3 | | NW. | Morning cloudy; afternoon and evening clear. |
| ☾ 26 | 29.720 | .030 | 65.7 | 9.7 | 26 | .206 | 35.2 | | NW. | Morning cloudy; afternoon and evening clear. |
| 27 | 29.681 | .070 | 70.0 | 4.3 | 39 | .330 | 47.6 | 0.039 | SE. | Cloudy; *rain* during the night. |
| 28 | 29.427 | .254 | 69.5 | 4.8 | 75 | .529 | 60.6 | 0.038 | (Var.) | Morning and aft. cloudy and *showery;* evening clear. *Bar. lowest* 29.392. |
| 29 | 29.493 | .071 | 69.0 | 4.5 | 40 | .349 | 49.1 | 0.283 | W. | Morning clear; afternoon and evening cloudy; 5 to 6, P.M., *rain.* |
| 30 | 29.715 | .222 | 54.0 | 15.0 | 46 | .216 | 36.5 | 0.010 | WNW. | Morning and evening clear; afternoon a *shower.* |
| 31 | 29.995 | .279 | 55.0 | 7.0 | 36 | .188 | 33.0 | | W. | Clear. *Thermometer lowest* 39°. Heavy hoar-frost; ice formed near the city. |
| Means for May, 1856 | 29.785 | [illegible] | 62.7 | 7.0 | 49 | .312 | 44.8 | 2.334 | N. 59° 37′ W. 21-100. | |
| Means for May, 5 yrs. | 29.843 | .110 | 63.6 | 5 8 | 50 | .367 | 50.3 | 4.175 | N. 70° 17′ W. 20-100. | |
| Means for the Spring, 1856 | 29.791 | .159 | 50.2 | 6.3 | 53 | .247 | 37.3 | 7.522 | N. 82° 6′ W. 31-100. | |
| Means for the Spring, 5 yrs. | 29.830 | .152 | 51.6 | 6.2 | 52 | .267 | 40.7 | 11.402 | N. 68° 41′ W. 24-100. | |

